# A large unruptured ectopic pregnancy

**DOI:** 10.1016/j.radcr.2021.02.048

**Published:** 2021-03-19

**Authors:** Metri Haddaden, Anil Maharaj, Kristofer Muzzi, Kalyan Paudel, Christopher J. Haas

**Affiliations:** a MedStar Health Internal Medicine, Baltimore, MD, USA; b Georgetown University School of Medicine, Washington, DC, USA; c MedStar Harbor Hospital, Department of Radiology, MD, USA

**Keywords:** Ectopic pregnancy, Large, Unruptured

## Abstract

Ectopic pregnancy carries a significant mortality risk in the first trimester given the increased likelihood of rupture of large embryos. In this report, an otherwise asymptomatic woman presented with amenorrhea and a positive urine pregnancy test. Results included an elevated beta-human chorionic gonadotropin (B-hCG) of 39,947 IU/L and Transvaginal ultrasound suggestive of a 3.9 cm unruptured fallopian tube ectopic pregnancy. She underwent emergent salpingectomy without complications, confirming a 10 week, 6 days gestational age pregnancy. This exceptional case of an unruptured ectopic with crown rump length (CRL) above 2 cm illustrates the importance of early diagnosis due to the often unremarkable presentation.

## Introduction

Ectopic pregnancy constitutes up to 1.4% of all reported pregnancies [1], and ruptured ectopic pregnancies remain a leading cause of maternal death in the first trimester, accounting for 15% of all mortalities [2]. While a fertilized egg can attach to numerous sites outside the uterus, 95% occur within the fallopian tubes [2]. Important risk factors associated with ectopic pregnancy include alterations in tubal anatomy, which may occur in the context of infection, previous tubal surgery, or tobacco use [3]. Symptoms of ectopic pregnancy generally manifest between 6 and 9 weeks of gestation and include a variety of nonspecific complaints - aching pelvic pain, abdominal discomfort with referred shoulder radiation, adnexal enlargement/tenderness, vaginal spotting, and amenorrhea [4]. Rupture and subsequent hemorrhage constitute the most significant complication of ectopic pregnancy, requiring emergent management. Here we present a case of a large, unruptured 10 week, 6 day ectopic pregnancy.

## Case presentation

A 34-year-old gravida 2 para 2 (0, 2, 0, 2) woman with a past medical history of sickle cell anemia presented to the emergency department with vaginal spotting, headache, and lightheadedness of a few days' duration. She was generally healthy and otherwise asymptomatic, with no noticeable menstrual irregularities until eight weeks prior to presentation, when she had a positive home pregnancy test. Both of her previous pregnancies were delivered prematurely at 20 and 21 weeks without any major maternal complications. She denied a history of sexually transmitted infections, new medications, substance use, or significant changes in medical care.

**Fig. 1:**
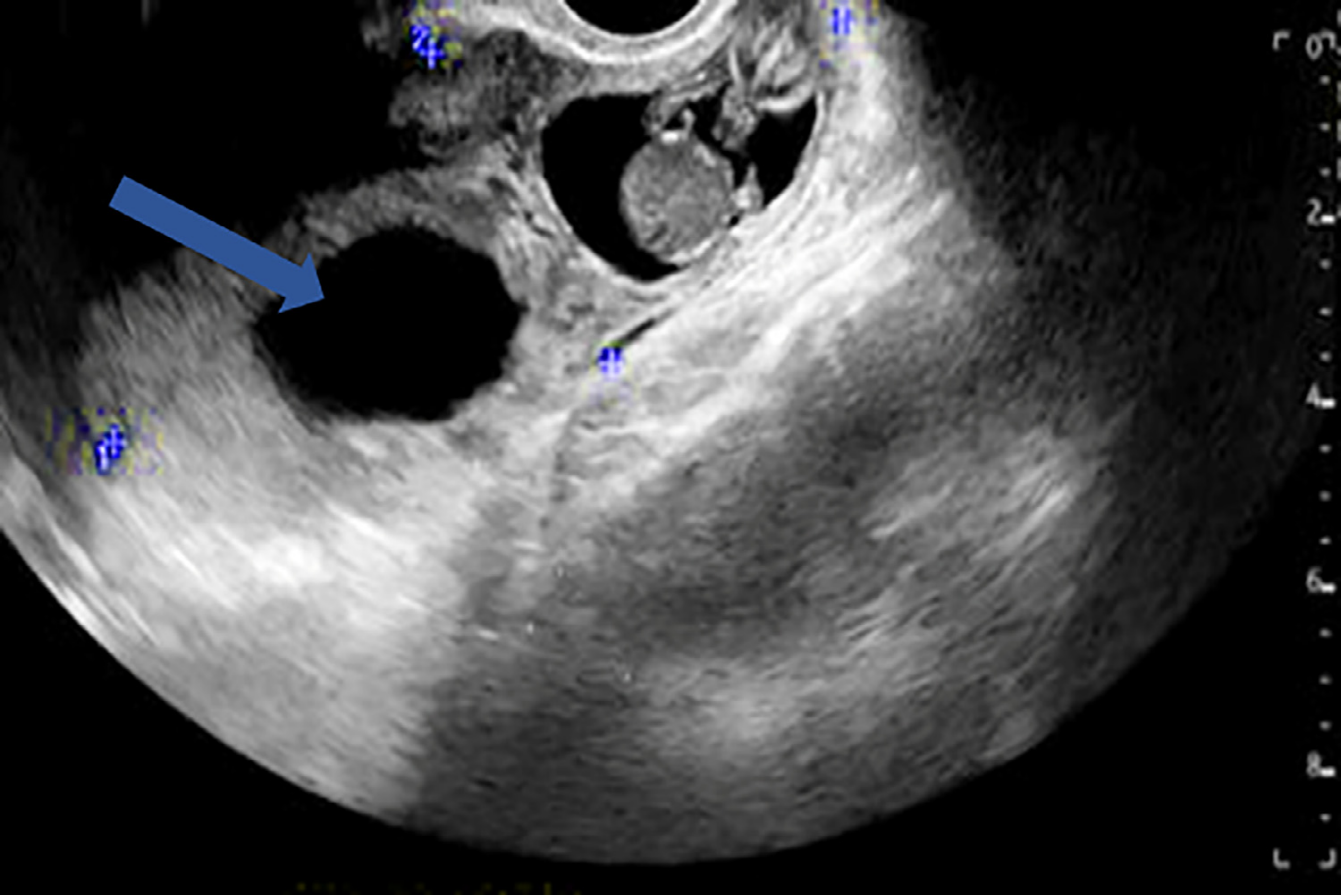
A transvaginal ultrasound demonstrating a large right adnexal mass measuring 10 cm × 4 cm containing an embryo and amniotic fluid (blue arrow).

**Fig. 2:**
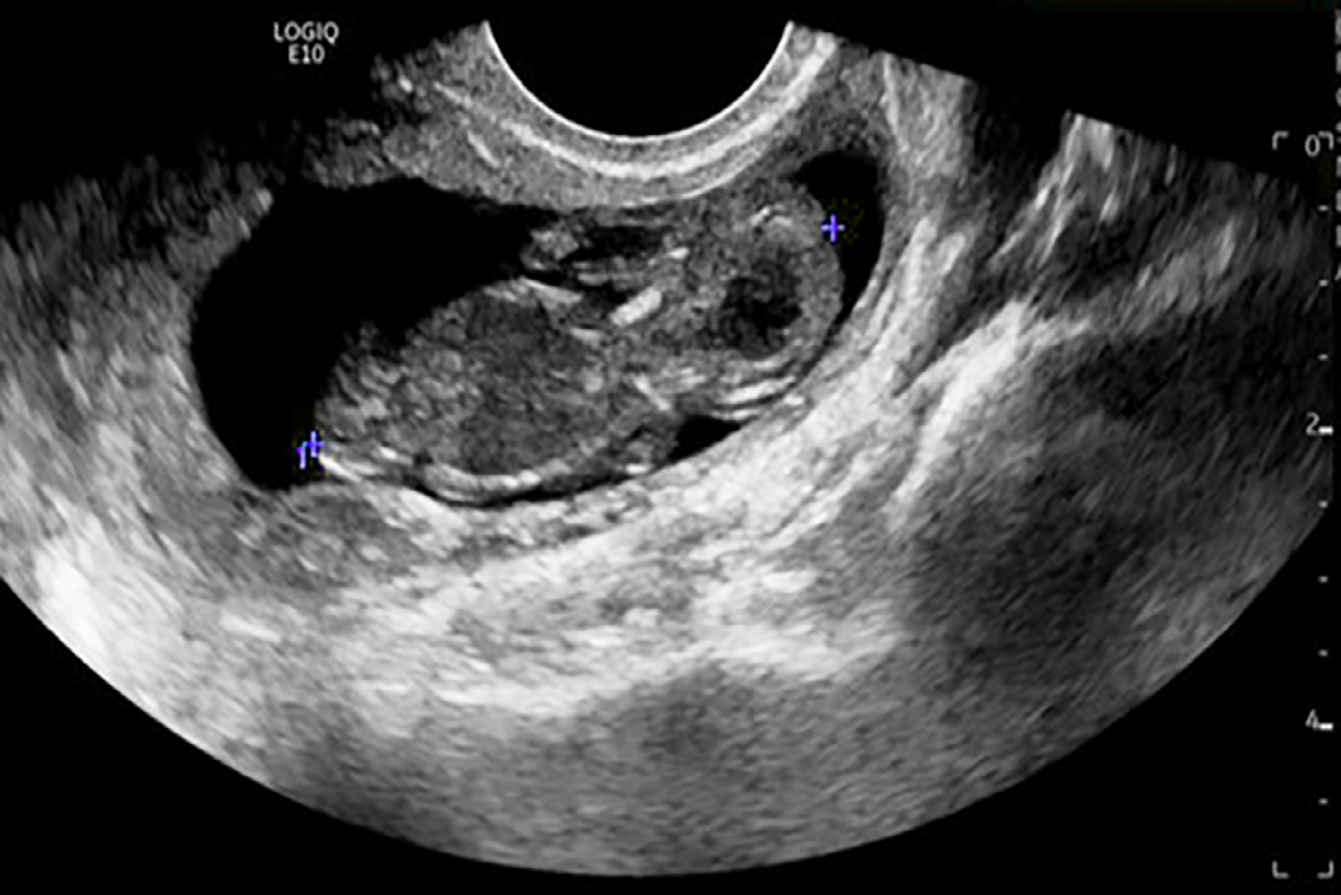
A transvaginal ultrasound demonstrating a large right adnexal mass containing an embryo with CRL of 3.9 cm corresponding to 10 weeks, 6 days gestational age.

Upon presentation to the emergency department, she remained hemodynamically stable. She was afebrile with a preserved heart rate (93 bpm), blood pressure (146/96 mmHg), and oxygen saturation on room air with a normal respiratory rate. Orthostatic vital signs were notably positive. Abdominal and vaginal examinations were both unremarkable, with no signs of vaginal spotting or adnexal masses. Complete blood count was significant for a stable, microcytic anemia (hemoglobin of 7.8 gm/dL, mean corpuscular volume of 62.9 FL) and an elevated red cell distribution width of 17.6%. Serum chemistries were unremarkable. B-hCG was elevated to 39,947 IU/L. Transabdominal and transvaginal ultrasound demonstrated an ectopic pregnancy measuring 10 cm × 4 cm (Fig. 1) containing a gestational sac and fetal pole with a crown rump length (CRL) measuring 3.9 cm (Fig. 2) within the right fallopian tube, an absence of fetal cardiac activity, and an enlarged uterus with heterogenous endometrium consistent with a non-viable ectopic pregnancy (Fig. 1). Obstetrics/Gynecology was consulted and recommended emergent laparoscopic salpingectomy. Postoperatively, her course was uneventful except for residual right sided abdominal pain. She was discharged the following day with oral pain medication and supplemental iron along with continued follow-up for birth control, counseling, and regular gynecologic care.

## Discussion

The diagnosis of ectopic pregnancy remains a clinical challenge that requires a high index of suspicion, particularly in premenopausal women with pertinent risk factors who present in the context of abdominal or pelvic pain, secondary amenorrhea, and vaginal bleeding. Physical examination, specifically adnexal tenderness and an enlarged uterus, in combination with an elevated B-hCG and pelvic ultrasound demonstrating a lack of intrauterine pregnancy confirm the diagnosis. Typically, serum quantitative B-hCG is expected to double every 2-3.5 days in the fourth to eighth weeks of gestation [2], with intrauterine pregnancies visualized by transvaginal ultrasound TVUS once B-hCG levels rise to the “discriminatory zone” of 1000-2000 IU/L [5]. Failure to visualize an intrauterine pregnancy at this time, along with other findings, such as an extrauterine mass and fluid in the pouch of Douglas, would confirm the diagnosis of ectopic pregnancy [4]. The average ectopic pregnancy size is 1.5 cm to 3.5 cm with an increased risk of rupture with increasing size [6], [7], [8]. Intriguingly, a B-hCG of more than 17,500 IU/L was shown to predict rupture with sensitivity of 62% and specificity of 73.6% [9]. Only a limited number of reports have demonstrated ectopic pregnancy with a CRL of more than 2 cm [4]. Here we report a case of a large, unruptured tubal ectopic pregnancy mass measuring 10 cm × 4 cm with a CRL of 3.9 cm and markedly elevated B-hCG of 39,947 IU/L.

The management of ectopic pregnancy depends on several factors - B-hCG level, gestational sac diameter, and hemodynamic stability. Expectant management is advised in ectopic pregnancy with a B-hCG level below 1000 IU/L, regardless of its size on ultrasound [9], whereas medical or surgical management is pursued if the B-hCG rises greater than 1000 IU/L. Methotrexate is recommended if patients meet the following criteria: B-hCG below 5000 IU/L, rising B-hCG level in 48 hours, normal hemoglobin, leukocytes, platelets, and liver enzymes, and a gestational sac diameter of 4 cm [4]. Surgical intervention is typically reserved for ruptured ectopic pregnancies, hemodynamical instability, and signs of an impending rupture in the setting of failure of medical therapy [4].

## Conclusion

It is typically thought that as ectopic pregnancy size increases, so too does symptom severity. However, as evidenced by our case of a nearly asymptomatic woman with a large, unruptured ectopic pregnancy, subjective presentation alone cannot be used as a predictor for ectopic pregnancy. A complete work-up, including B-hCG and imaging, along with Obstetrics consultation, is critical in preventing the risk for worsening complications and potential maternal mortality.
