# Erratum to “A large unruptured ectopic pregnancy” [Radiology Case Reports 16 (2021) 1204–1206]

**DOI:** 10.1016/j.radcr.2023.01.044

**Published:** 2023-02-22

**Authors:** Metri Haddaden, Anil Maharaj, Kristofer Muzzi, Kalyan Paudel, Christopher J. Haas

**Affiliations:** a MedStar Health Internal Medicine, Baltimore, MD, USA; b Georgetown University School of Medicine, Washington, DC, USA; c Department of Radiology, MedStar Harbor Hospital, Baltimore, MD, USA

This article was published without appropriate declaration of competing interest or patient consent statements.

The publisher apologizes for any inconvenience caused by this mistake. The relevant statements are provided below.

**Declaration of Competing Interest**

The authors declare that they have no known competing financial interests or personal relationships that could have appeared to influence the work reported in this paper.

**Patient consent**

The authors have written informed consent from the patient for the publication of this case report.

